# De Novo Actionable Genomic Alterations in High-Grade Pulmonary Neuroendocrine Carcinomas: Therapeutic Implications of Targeted Treatment

**DOI:** 10.3390/medsci14040491

**Published:** 2026-08-18

**Authors:** Ari Raphael, Nir Peled, Roni Gillis, Hovav Nechushtan, Walid Shalata, Elizabeth Dudnik

**Affiliations:** 1 Davidoff Cancer Center, Rabin Medical Center, Petah Tikva 4941492, Israel; 2 Gray Faculty of Medicine, Tel Aviv University, Tel Aviv 6997801, Israel; 3 Helmsley Cancer Center, Shaare Zedek Medical Center, Jerusalem 9103102, Israel; nirp@szmc.org.il (N.P.); ronigil@szmc.org.il (R.G.); 4 Faculty of Medicine, The Hebrew University of Jerusalem, Jerusalem 9112102, Israel; hovavnech@hadassah.org.il; 5 Sharett Institute of Oncology, Hadassah Medical Center, Jerusalem 9112001, Israel; 6 The Legacy Heritage Cancer Center and Dr. Larry Norton Institute, Soroka University Medical Center, Beer-Sheva 8410101, Israel; walid_sh@clalit.org.il; 7 Joyce & Irving Goldman Medical School, Ben-Gurion University of the Negev, Beer-Sheva 8410501, Israel; elizabeth.dudnik1603@gmail.com; 8 Lung Cancer Service, Assuta Medical Centers, Tel Aviv 6971028, Israel

**Keywords:** high-grade neuroendocrine carcinoma, LCNEC, SCLC, targeted therapy, *EGFR*, *ALK*

## Abstract

Background/Objectives: High-grade pulmonary neuroendocrine carcinoma (HGNEC-L), including SCLC and LCNEC, is aggressive and usually treated according to SCLC paradigms. The clinical relevance of de novo actionable genomic alterations (AGA) remains incompletely defined. Methods: We performed a retrospective multicenter analysis of advanced HGNEC-L with de novo AGA, assessing rwORR, rwDCR, rwPFS, and OS. Findings were contextualized by a structured literature review and exploratory pooled reconstructed-IPD Cox analysis, with targeted therapy modeled as a source-stratified time-dependent covariate. Results: Ten patients from four tertiary centers were included. Most were women (90.0%) and never-smokers (70.0%); histology was LCNEC in 60.0% and SCLC/mixed SCLC in 40.0%. AGA included *EGFR* mutations (*n* = 6), *EML4*-*ALK* fusions (*n* = 2), *KIF5B*-*RET* fusion (*n* = 1), and *KRAS* p.G12C (*n* = 1). Targeted-containing regimens achieved rwORR 77.8%, rwDCR 88.9%, and median rwPFS 9.0 months (95% CI, 2.0–18.7), as opposed to 3.7 months for ICI-containing regimens and 2.6 months for chemotherapy alone. Median OS for the entire cohort was 17.2 months (95% CI, 4.8–36.3); overall survival did not differ significantly between patients with and without targeted-containing exposure (19.0 vs. 17.2 months; log-rank *p* = 0.50). In pooled reconstructed-IPD time-dependent Cox analysis (72 patients, 39 deaths), targeted therapy showed a favorable but non-significant OS association (HR, 0.89; 95% CI, 0.31–2.56; *p* = 0.831), maintained directionally in the age/sex-adjusted subset (HR, 0.54; 95% CI, 0.16–1.77; *p* = 0.306). Conclusions: De novo AGA-positive HGNEC-L represents a clinically relevant subgroup with potential sensitivity to genotype-matched targeted therapy, supporting comprehensive molecular profiling and early targeted therapy consideration.

## 1. Introduction

Lung neuroendocrine neoplasms comprise a biologically heterogeneous spectrum that includes low-grade typical and atypical carcinoids and high-grade neuroendocrine carcinomas, primarily small-cell lung cancer (SCLC) and large-cell neuroendocrine carcinoma (LCNEC) [1,2]. High-grade pulmonary neuroendocrine carcinoma (HGNEC-L) is characterized by rapid proliferation, early dissemination, and poor prognosis. Historically, systemic therapy for advanced HGNEC-L has relied largely on platinum-etoposide-based regimens adapted from SCLC, with limited incorporation of molecularly selected treatment strategies [2,3].

Comprehensive genomic profiling has challenged the view of HGNEC-L as a uniformly SCLC-like entity. In LCNEC, genomic studies have identified at least two major molecular patterns: a SCLC-like subtype enriched for concurrent *TP53* and *RB1* alterations, and an NSCLC-like subtype characterized by genomic features more commonly associated with non-small cell lung cancer, including alterations in *STK11*, *KEAP1*, *KRAS*, and other potentially targetable oncogenic drivers [3,4,5]. 

De novo actionable genomic alterations (AGA) in HGNEC-L are uncommon, but their detection has increasing therapeutic implications. Reported alterations include activating *EGFR* mutations, *ALK* and *RET* rearrangements, *BRAF* p.V600E, *KRAS* p.G12C, *MET* exon 14 alterations, *ROS1* fusions, and other rare kinase drivers [6,7,8]. Early reports of *EGFR*-mutant SCLC and combined SCLC with adenocarcinoma demonstrated that *EGFR* mutations, although uncommon in SCLC, may occur in never- or light-smokers and in tumors with combined adenocarcinoma/SCLC components [9,10,11]. However, patients with HGNEC-L have generally been underrepresented or excluded from pivotal genotype-directed trials in lung cancer. 

Published experience suggests that a subset of de novo AGA-positive HGNEC-L can achieve clinically meaningful responses to matched targeted therapy [12,13,14,15,16,17,18,19,20,21,22,23,24,25,26,27,28,29,30,31,32,33,34,35,36,37,38,39,40]. Nevertheless, interpretation is complicated by several factors: variable histologic definitions, mixed LCNEC/SCLC or combined tumors, inconsistent reporting of treatment sequence, incomplete patient-level survival data, and publication bias favoring exceptional responders. These limitations have restricted the ability to estimate the association between targeted therapy exposure and survival.

To address this gap, we performed a retrospective multicenter cohort analysis of patients with advanced-stage HGNEC-L harboring de novo AGA detected at baseline. We then contextualized the institutional cohort through a structured review of published systemic treatment outcomes in de novo AGA-positive HGNEC-L. Finally, to reduce immortal-time bias and account for delayed initiation of targeted therapy, we performed an exploratory pooled reconstructed source-stratified time-dependent individual-patient data Cox analysis combining our cohort with published cases reporting patient-level survival data. The aim of this study was to evaluate the therapeutic implications of targeted therapy in advanced HGNEC-L with de novo AGA and to define the limitations of the currently available evidence base. Compared with the recent European LCNEC series reported by Heijboer et al. [12], the present cohort extends the available evidence to SCLC and mixed high-grade neuroendocrine histologies, incorporates less frequently reported drivers such as *KRAS* p.G12C and *EGFR* exon 20 insertion, and derives from a demographically distinct, markedly never-smoker- and female-enriched population, thereby broadening the spectrum of de novo AGA-positive HGNEC-L in which genotype-matched therapy may be clinically relevant.

## 2. Materials and Methods

### 2.1. Study Design

#### Retrospective Multicenter Cohort Analysis

We performed a retrospective multicenter analysis of patients with advanced-stage HGNEC-L harboring de novo AGA. Patients were treated across four tertiary centers between 2018 and 2024. Eligible cases had histologically confirmed advanced-stage HGNEC-L, including SCLC, LCNEC, or combined high-grade neuroendocrine histologies, together with identification of a baseline actionable Tier 1 genomic alteration by next-generation sequencing (NGS) prior to the initiation of any systemic therapy. De novo alterations were defined as those identified at baseline molecular testing performed before any systemic therapy for advanced disease. Prior surgical resection or adjuvant chemotherapy for earlier-stage disease was permitted, whereas cases with prior tyrosine kinase inhibitor exposure or documented histologic transformation from non-small cell lung cancer were excluded. Molecular testing was performed using validated clinical platforms, including Oncomine Dx (Thermo Fisher Scientific, Waltham, MA, USA), Tempus xT (Tempus AI, Chicago, IL, USA), FoundationOne CDx (Foundation Medicine, Cambridge, MA, USA), and Guardant360 (Guardant Health, Redwood City, CA, USA). Clinical, pathologic, molecular, treatment, and outcome data were extracted retrospectively from institutional records. Histologic diagnoses of SCLC, LCNEC, and combined high-grade neuroendocrine carcinoma were established locally according to the WHO classification of thoracic tumors, integrating morphology with neuroendocrine immunohistochemical markers (e.g., synaptophysin, chromogranin A, INSM1, and Ki-67 proliferation index); central pathology re-review was not performed.

The main outcomes of the cohort analysis were real-world objective response rate (rwORR), real-world disease control rate (rwDCR), real-world progression-free survival (rwPFS), and overall survival (OS) across systemic treatment classes, with particular emphasis on targeted-containing regimens. rwORR was defined as the proportion of evaluable treatment lines with a documented complete or partial response, and rwDCR as the proportion with complete response, partial response, or stable disease. rwPFS was defined as the time from treatment initiation to radiographic progression or death from any cause. OS was defined as the time from first-line systemic treatment initiation for advanced disease to death from any cause.

### 2.2. Literature Search and Review

To contextualize the institutional cohort, we conducted a structured literature review on the efficacy of systemic treatment in HGNEC-L with de novo AGA. The search focused on advanced-stage pulmonary SCLC, LCNEC, and combined high-grade neuroendocrine carcinomas in which an actionable driver was identified at diagnosis or before the start of systemic therapy. We prioritized reports providing actual treatment efficacy data, including radiographic response, progression-free survival, or overall survival.

Eligible publications included case reports, case series, and retrospective cohorts describing de novo advanced HGNEC-L with actionable genomic alterations and reporting treatment outcomes. Reports were excluded if they described histologic transformation from non-small cell lung cancer, acquired resistance alterations rather than baseline drivers, non-pulmonary neuroendocrine carcinomas, or cases lacking treatment efficacy data. For eligible reports, available patient-level data were extracted on clinicopathologic features, genomic alterations, treatment exposure, and survival outcomes. 

### 2.3. Exploratory Pooled Individual-Patient Analysis

We subsequently performed an exploratory pooled reconstructed source-stratified time-dependent individual-patient data-based Cox analysis combining our cohort with published cases that reported patient-level OS data. The purpose of this analysis was to explore the association between exposure to targeted therapy and survival in patients with advanced HGNEC-L harboring de novo AGA. Extracted variables included age, sex, treatment sequence, timing of targeted therapy exposure, and OS. When individual patient data were not directly reported but could be extracted from published patient-level timelines, reconstructed individual patient data were generated and incorporated. To reduce bias related to differences in the timing of treatment initiation, targeted therapy exposure was handled as a time-dependent variable. Accordingly, patients contributed follow-up time to the non-exposed group from the start of first-line systemic therapy until the initiation of targeted therapy, if such treatment was subsequently given, and contributed follow-up time to the targeted-therapy-exposed group thereafter. Patients who never received targeted therapy remained in the non-exposed group throughout follow-up. 

### 2.4. Statistical Analysis

Statistical analyses were performed using Python, version 3.11.2. Categorical variables were summarized as counts and percentages, and continuous variables were described using medians and ranges. RwORR and rwDCR were calculated descriptively across treatment classes and grouped treatment categories. RwPFS and OS were estimated using the Kaplan–Meier method, and median survival times were reported with 95% confidence intervals (CIs). For the exploratory pooled individual-patient survival analysis, we combined our institutional cohort with published cases that provided usable patient-level overall survival or follow-up information. Overall survival was analyzed from the start of first-line systemic therapy to death from any cause, with censoring at last reported follow-up. To account for variation in the timing of targeted treatment initiation and to avoid immortal-time bias, targeted-therapy exposure was modeled as a time-dependent covariate in a Cox proportional hazards model. Because the pooled dataset included patients from different data sources, analyses were stratified by source, allowing for source-specific baseline hazards. A sensitivity analysis was performed in the subset of patients with exact age and sex data available, in which the model was additionally adjusted for age and sex.

## 3. Results

### 3.1. Cohort Characteristics and Treatment Outcomes

#### 3.1.1. Baseline Patient, Tumor and Treatment Characteristics

The cohort comprised ten patients with advanced-stage HGNEC-L harboring de novo AGA. The median age at diagnosis of advanced disease was 64.6 years (range, 47.1–77.0). The cohort was markedly enriched for female patients (90.0%) and never-smokers (70.0%), with a median smoking exposure of 0 pack-years (range, 0–20). No patient had a reported family history of lung cancer, and most patients had a good performance status, with ECOG PS 0–1 in 90.0% of cases. The single patient with ECOG performance status 3, who harbored an *EGFR* exon 19 deletion with *TP53* co-mutation, received first-line chemo-immunotherapy (pembrolizumab plus platinum-etoposide) with a partial response and a progression-free interval of approximately 13 months before second-line chemotherapy, indicating that the poor baseline performance status did not preclude meaningful first-line benefit; given the cohort size, a formal sensitivity analysis excluding this single patient was not statistically informative. Histologically, 60% of tumors were classified as large-cell neuroendocrine carcinoma, while the remaining 40% represented small-cell or mixed neuroendocrine carcinoma (Table 1A).

The molecular landscape was dominated by *EGFR* alterations, identified in six patients, including five exon 19 deletions and one exon 20 insertion. The single *EGFR* exon 20 insertion case, a combined SCLC/adenocarcinoma with brain metastases, received first-line platinum-etoposide chemotherapy with a partial response and died approximately five months after first-line initiation; a matched exon 20-directed agent was not administered. Consistent with Zullo et al. [33], classical *EGFR*-directed agents have limited activity against *EGFR* exon 20 insertions, and this case was therefore analyzed separately from the classical-*EGFR* (exon 19 deletion and p.L858R) cases. Gene rearrangements were observed in three patients, including two cases with *EML4*-*ALK* fusion and one case with *KIF5B*-*RET* fusion. One patient harbored a *KRAS* p.G12C mutation. Co-mutations in *TP53* and *RB1* were identified in 60% and 30% of cases, respectively. Biomarker assessment was limited, with PD-L1, TMB, and MSI data available in 30.0%, 40.0%, and 40.0% of cases, respectively; all evaluable MSI results were MSI-stable (Table 1B).

The treatment landscape was heterogeneous but notably enriched for targeted-based approaches (Table 1C). Across all 19 treatment lines, targeted therapy was the most frequently used individual treatment class, accounting for 31.6% of all lines, with 50.0% of patients receiving targeted therapy at any point and 30.0% receiving it in the first-line setting. Combination of chemotherapy with targeted therapy represented an additional 15.8% of all treatment lines, was administered to 20.0% of patients overall, and was used in the first line in 20.0% of cases. By contrast, chemotherapy alone accounted for 26.3% of all treatment lines, with 50.0% of patients ever exposed and 20.0% treated with chemotherapy alone in the first line. Combination of chemotherapy with ICI comprised 15.8% of all treatment lines and was used exclusively in the first-line setting, where it accounted for 30.0% of initial treatments. When treatment classes were grouped, targeted-containing regimens emerged as the dominant therapeutic strategy, comprising 47.4% of all treatment lines, with 70.0% of patients exposed overall and 50.0% receiving a targeted-containing regimen as first-line treatment. In comparison, ICI-containing regimens accounted for 21.1% of all treatment lines and were administered to 40.0% of patients overall, while ADC exposure was limited to a single later-line treatment. Overall, in this cohort of high-grade pulmonary neuroendocrine carcinomas harboring de novo actionable genomic alterations, targeted-containing treatment constituted a major component of the therapeutic landscape and was incorporated early in the disease course in a substantial proportion of patients.

#### 3.1.2. Treatment Efficacy

Targeted-based regimens demonstrated encouraging antitumor activity in this cohort. Among individual treatment classes, targeted therapy achieved a rwORR of 66.7% and a rwDCR of 83.3%, while chemo-targeted therapy achieved a rwORR and rwDCR of 100.0% each; by comparison, chemotherapy alone was associated with a rwORR and rwDCR of 25.0%, and single-agent ICI or ADC showed no objective responses or disease control in the small number of evaluable cases (Table 2A). When treatment classes were grouped, targeted-containing regimens yielded the most favorable overall activity, with a rwORR of 77.8% and a rwDCR of 88.9%, compared with 66.7% and 66.7%, respectively, for ICI-containing regimens and 25.0% and 25.0% for chemotherapy only (Table 2B). In the first-line setting, targeted-containing regimens achieved a rwORR of 60.0% and a rwDCR of 80.0%, whereas ICI-containing regimens and chemotherapy only were each associated with a rwORR and rwDCR of 100.0%, although these estimates were based on only two and one evaluable patients, respectively (Table 2C). 

**Figure 1:**
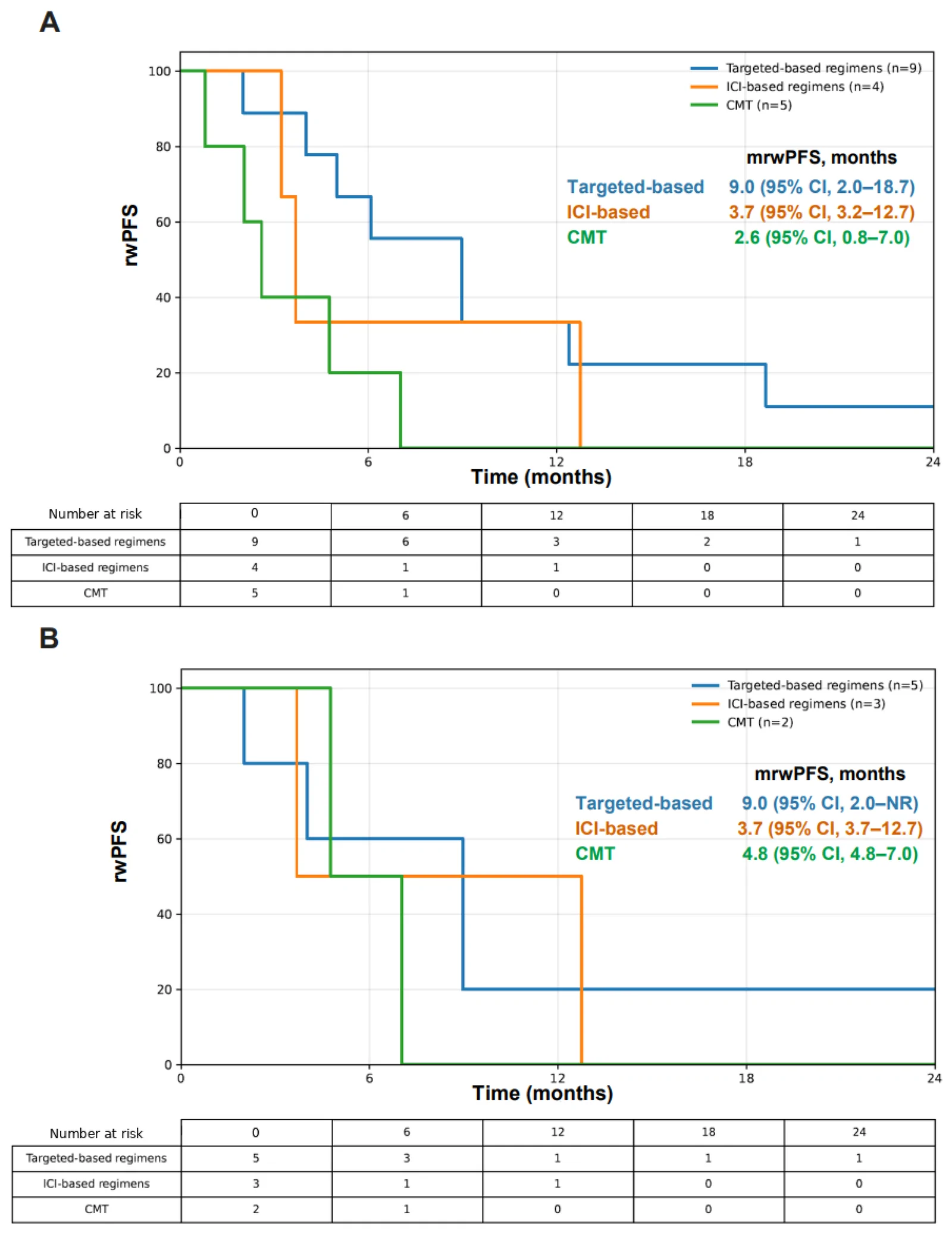
Real-world progression-free survival with targeted-containing regimens, ICI-containing regimens, and CMT given as any treatment line (**A**) and given as a first-line treatment (**B**).

Consistent with the response data, rwPFS was longest with targeted-based regimens: across all treatment lines, median rwPFS was 9.0 months (95% CI, 2.0–18.7) for targeted-based regimens, compared with 3.7 months (95% CI, 3.2–12.7) for ICI-based regimens and 2.6 months (95% CI, 0.8–7.0) for chemotherapy alone (Figure 1A). A similar pattern was observed in the first-line setting, where median rwPFS was 9.0 months (95% CI, 2.0–NR) with targeted-based regimens, 3.7 months (95% CI, 3.7–12.7) with ICI-based regimens, and 4.8 months (95% CI, 4.8–7.0) with chemotherapy only (Figure 1B). Among the three patients with baseline brain metastases, intracranial responses were documented in two: a partial intracranial response in a patient with *KIF5B*-*RET* fusion treated with first-line pralsetinib, and a partial intracranial response in the combined-SCLC *EGFR* exon 20 insertion patient; intracranial response data were unavailable for the third patient.

### 3.2. OS Analysis

**Figure 2:**
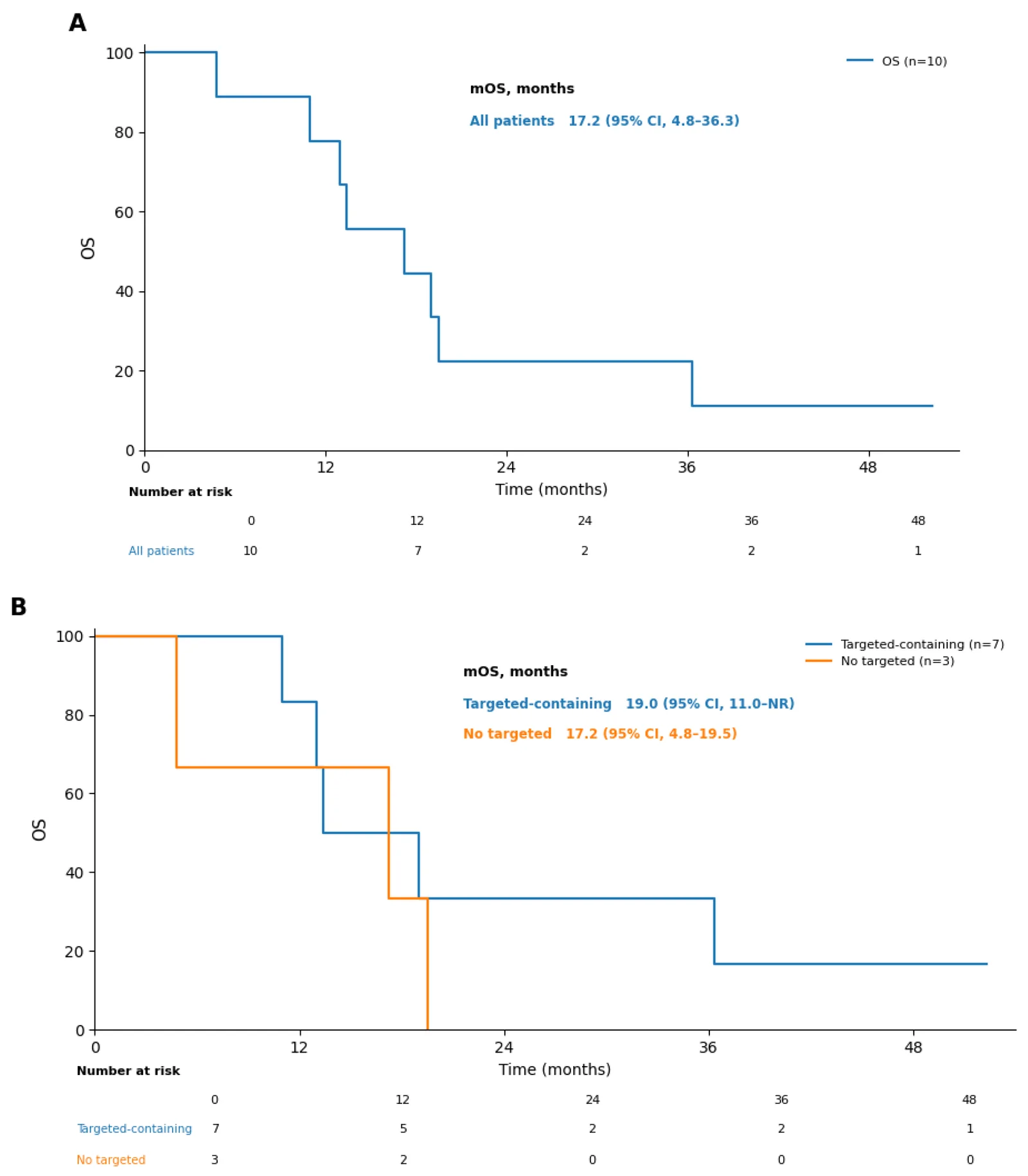
Overall survival (**A**) in all patients (*n* = 10) and (**B**) in accordance with the exposure to targeted-containing regimens.

Overall survival was assessed in all ten patients. The median overall survival for the entire cohort was 17.2 months (95% CI, 4.8–36.3), with eight deaths and two censored patients (Figure 2A). When patients were grouped by exposure to a targeted-containing regimen at any time, median overall survival was 19.0 months (95% CI, 11.0–not reached) in exposed patients (*n* = 7) versus 17.2 months (95% CI, 4.8–19.5) in unexposed patients (*n* = 3); this difference was not statistically significant (log-rank *p* = 0.50). Given the very small subgroups, these overall survival estimates are presented descriptively and should not be interpreted as evidence of a survival benefit (Figure 2B). 

### 3.3. Literature Review Findings

We performed a literature search focused on advanced-stage high-grade neuroendocrine lung cancers, including SCLC, LCNEC, and HGNEC-L, harboring de novo actionable driver alterations identified at diagnosis. Priority was given to reports providing treatment efficacy data. For eligible publications, available patient-level information was extracted, including clinicopathologic characteristics, genomic alterations, treatment exposure, and survival outcomes. The published evidence remains limited and consists predominantly of case reports and small retrospective series, with most reported cases involving LCNEC. The most consistent signal of clinical benefit was observed with matched targeted therapy in *EGFR*-, *ALK*-, and *RET*-driven LCNEC (Table 3).

### 3.4. Exploratory Pooled Individual-Patient Cox Model OS Analysis 

We subsequently performed an exploratory pooled reconstructed individual-patient data analysis using a source-stratified, time-dependent Cox model, combining our cohort with published cases for which patient-level OS data were available. Based on the pooled reconstructed-IPD dataset (Supplementary Table S1), the primary analysis included 72 analyzable patients and 39 death events. Ito et al. (2017) [26] and Ricciuti et al. (2018) [28] were excluded because exact patient-level efficacy data were not available. Targeted-therapy exposure was modeled as a time-dependent covariate and was not significantly associated with OS in the primary source-stratified model (HR, 0.89; 95% CI, 0.31–2.56; *p* = 0.831; Table 4).

A sensitivity analysis restricted to patients with exact age and sex data included 46 patients and 18 death events, with additional adjustment for age and sex. In this model, the point estimate remained directionally favorable for targeted-therapy exposure but did not reach statistical significance (HR, 0.54; 95% CI, 0.16–1.77; *p* = 0.306; Table 4). Age, modeled per 10-year increase, was not significantly associated with OS (HR, 0.90; 95% CI, 0.58–1.40; *p* = 0.633), nor was male sex (HR, 1.17; 95% CI, 0.28–4.96; *p* = 0.828; Table 4).

This analysis yielded a hazard ratio below 1, supporting a positive directional signal for targeted therapy exposure, although the estimate remained highly imprecise. This imprecision likely reflects the heterogeneity of the pooled reports, the retrospective and reconstructed nature of the case-level data, and the limited number of death events, particularly in the exact-age sensitivity subset.

## 4. Discussion

In this retrospective institutional multicenter cohort, advanced-stage HGNEC-L harboring de novo AGA showed encouraging real-world response and progression-free survival signals with genotype-matched therapy, without a demonstrable difference in overall survival. The cohort was enriched for clinicopathologic features often associated with oncogene-driven lung cancer, including female sex and never-smoking status. Despite high-grade neuroendocrine histology, targeted-containing regimens produced the most favorable outcomes across treatment classes, with a rwORR of 77.8%, rwDCR of 88.9%, and median rwPFS of 9.0 months. Overall survival did not differ significantly between patients with and without targeted-containing exposure (19.0 versus 17.2 months; log-rank *p* = 0.50), and given the small subgroups this comparison is descriptive only. These results support the concept that, in at least a subset of HGNEC-L, oncogenic driver dependence may remain therapeutically dominant even in the presence of aggressive neuroendocrine morphology. This point is clinically relevant because HGNEC-L has traditionally been approached as a biologically uniform, rapidly proliferative disease, often managed according to SCLC-based paradigms. Our findings suggest that histologic classification alone may be insufficient to guide systemic treatment in driver-positive cases. Rather, the coexistence of high-grade neuroendocrine differentiation and an AGA may define a biologically distinct subgroup in which molecular selection can meaningfully influence treatment sensitivity and disease trajectory.

Within our institutional cohort, ICI-containing regimens showed shorter median rwPFS than targeted-containing regimens despite some objective responses in the first-line setting. This finding should be interpreted cautiously because of the very small number of evaluable treatment lines, but it is consistent with broader observations that oncogene-driven lung cancers may be less responsive to immune checkpoint blockade than smoking-associated tumors without canonical drivers [8]. Importantly, the present data do not exclude a role for immunotherapy in HGNEC-L, particularly in clinically aggressive presentations, high-volume disease, or in tumors with molecular features more typical of smoking-associated neuroendocrine carcinoma. However, the observed pattern raises concern that ICI-based treatment may not be the optimal default strategy for patients whose tumors harbor AGA. In this setting, treatment decisions should integrate smoking history, pace of the disease, clinically meaningful symptom burden or lack thereof, molecular profile including molecular co-mutational status, and available immune biomarkers, while recognizing that PD-L1 expression, tumor mutational burden, and other predictors remain incompletely characterized in rare driver-positive HGNEC-L.

The observed phenomenon is consistent with the evolving molecular taxonomy of LCNEC. Prior genomic studies demonstrated that LCNEC is not a single molecular entity but includes tumors with SCLC-like and NSCLC-like genomic architecture [3,4,5]. The presence of actionable drivers such as *EGFR* mutations and *ALK* rearrangements is more compatible with NSCLC-like biology, although these alterations can coexist with *TP53* and *RB1* abnormalities and with morphologic neuroendocrine differentiation [3,4,5,6,7]. In the present cohort, *TP53* and *RB1* co-mutations were observed in 60.0% and 30.0% of cases, respectively. Importantly, these co-alterations did not preclude responses to targeted therapy, suggesting that driver-directed treatment should not be withheld solely because of high-grade neuroendocrine histology or the presence of aggressive co-mutational features. This is particularly important because *TP53* and *RB1* alterations are often interpreted as markers of lineage plasticity and therapeutic resistance. Our data support a more nuanced interpretation: *TP53*/*RB1* disruption may contribute to neuroendocrine phenotype and aggressive behavior, but it does not necessarily extinguish dependence on the initiating or dominant oncogenic driver. This distinction may explain why some patients with HGNEC-L can achieve clinically meaningful responses to targeted agents despite adverse histologic and molecular features.

The literature focusing on the pathogenesis of HGNEC-L provides important context for interpreting the clinical phenotype observed in our cohort. Zakowski et al. first highlighted *EGFR*-mutant SCLC in never-smokers, while Fukui et al. and Tatematsu et al. linked *EGFR*-mutant SCLC to combined adenocarcinoma/SCLC histology and light- or never-smoking status [9,10,11]. These observations support the concept that at least some AGA-positive HGNEC-L may arise from, or share lineage with, adenocarcinoma-like oncogene-driven tumors rather than representing canonical smoking-associated SCLC biology. They also emphasize the importance of distinguishing de novo baseline AGAs from acquired histologic transformation after exposure to TKIs. This distinction is not merely semantic. Acquired SCLC transformation after targeted therapy represents a recognized resistance mechanism, whereas de novo AGA-positive HGNEC-L presents at baseline with both neuroendocrine morphology and a targetable driver. These two clinical entities may differ in clonal architecture, drug sensitivity, and expected patterns of resistance. Therefore, documenting whether the actionable alteration was present before systemic therapy is essential for interpreting outcomes and for avoiding inappropriate extrapolation from post-TKI transformation biology.

The structured literature review further supports the therapeutic relevance of de novo AGA in HGNEC-L. Across the eligible reports, most published cases involved LCNEC, and the most consistent activity was observed with matched targeted therapy in *ALK*-, *EGFR*-, and *RET*-driven tumors [12,13,14,15,16,17,18,19,20,21,22,23,24,25,26,27,28,29,30,31,32,33,34,35,36,37,38,39,40]. Several reports described durable disease control or prolonged survival with *ALK* TKIs, *EGFR* TKIs, *RET* TKIs, and *BRAF*/MEK inhibition in de novo HGNEC-L, whereas chemo-immunotherapy outcomes were more heterogeneous. Although individual reports cannot establish comparative efficacy, consistent responses across independent publications strengthen the biological plausibility of targeted-therapy sensitivity in this rare subgroup. The signal is not confined to a single alteration, drug class, or institution, but appears across several oncogene-defined subsets. Instead, the literature review suggests that when a clear oncogenic dependency is present, HGNEC-L may retain a therapeutically actionable component despite neuroendocrine differentiation. At the same time, the evidence remains fragmentary, and the published experience is inevitably influenced by selective reporting of unusual or favorable cases. In aggregate, the actionable alterations identified across the present cohort and the reviewed literature comprised *EGFR* mutations (exon 19 deletions, p.L858R, and exon 20 insertion), *ALK* and *RET* rearrangements, and less common *KRAS* p.G12C, *BRAF* p.V600E, *ROS1*, and *MET*-related drivers, with the most consistent evidence of benefit from genotype-matched targeted therapy observed in *EGFR*-, *ALK*-, and *RET*-driven tumors (summarized in Table 3).

The pooled reconstructed-IPD Cox analysis was designed to evaluate whether the apparent benefit of targeted therapy persisted after accounting for treatment timing. Because patients who receive targeted therapy later in the disease course must survive long enough to receive it, a conventional ever-versus-never comparison is vulnerable to immortal-time bias. Modeling targeted therapy exposure as a time-dependent covariate partially addresses this problem by assigning person-time before targeted therapy to the non-exposed state and person-time after targeted therapy initiation to the exposed state. In the primary source-stratified analysis including 72 patients and 39 death events, targeted-therapy exposure yielded a hazard ratio below 1 but was not statistically significant (HR, 0.89; 95% CI, 0.31–2.56; *p* = 0.831). In the sensitivity analysis restricted to patients with exact age and sex data, the point estimate was more favorable but remained imprecise (HR, 0.54; 95% CI, 0.16–1.77; *p* = 0.306). These results are best interpreted as directionally supportive but statistically inconclusive. The use of source stratification was intended to reduce confounding from differences between the institutional cohort, the Heijboer series [12], and individual case reports; however, stratification cannot correct for all differences in eligibility, treatment access, clinical selection, follow-up, and reporting details. The wide confidence intervals indicate that the available data are compatible with a range of possible effect sizes, including meaningful or limited benefit.

The non-significant pooled Cox result should not be interpreted as evidence against targeted therapy. Rather, it reflects limitations of the available data structure. The pooled dataset combined a small institutional cohort with heterogeneous reports, many of which were case reports selected for clinical interest or exceptional response. The Heijboer series [12] contributed a substantial number of targeted-treated patients, improving the description of outcomes among exposed patients, but because it was heavily enriched for targeted therapy, it provided limited within-source contrast between exposed and non-exposed follow-up. In addition, several published cases required reconstruction of OS and treatment timing from figures or narrative timelines, and some potentially relevant reports lacked sufficient patient-level survival anchors. Consequently, the model is methodologically appropriate but underpowered and vulnerable to imprecision, residual confounding, and publication bias. Another important issue is treatment sequencing. Patients who receive targeted therapy in later lines may have different disease biology and performance status than those treated upfront, while patients never exposed to targeted therapy may include individuals who deteriorated rapidly before molecular results or drug access became available. These real-world mechanisms can bias estimates in either direction and are difficult to resolve without prospective data collection.

These findings have practical clinical implications. First, comprehensive molecular profiling should be considered in advanced HGNEC-L, particularly in LCNEC, combined histologies, never-smokers, patients with adenocarcinoma-like clinical features, and cases with atypical molecular or clinical behavior. Second, when a de novo AGA is identified, early use of genotype-matched therapy should be considered, either alone or in rational combination with chemotherapy, depending on the driver, tumor burden, pace of disease, and clinical context. In routine practice, this means that tissue acquisition should be planned to support both diagnostic classification and broad genomic testing whenever feasible. Plasma-based testing may be useful when tissue is limited or when rapid therapeutic decision-making is required, but negative plasma results should be interpreted cautiously in the absence of adequate tissue profiling. Multidisciplinary review may also be valuable, because classification of HGNEC-L can be challenging, especially in small biopsies, mixed tumors, or cases with overlapping adenocarcinoma and neuroendocrine features. Where available, integration of morphology, immunohistochemistry, and genomic data may help identify patients who are most likely to benefit from genotype-matched treatment.

This study has several limitations. The institutional cohort was small, retrospective, and heterogeneous with respect to histology, driver alteration, treatment sequence, and line of therapy. Because the cohort was defined by the presence of an actionable driver, it was markedly enriched for women and never-smokers and does not reflect the typical demographic of high-grade neuroendocrine carcinoma, which usually arises in older patients with a heavy smoking history; the genomic and outcome data are therefore susceptible to selection bias and limited generalizability. Response assessment was based on real-world documentation rather than centralized radiologic review. Biomarker data such as PD-L1 expression, tumor mutational burden, and MSI status were incomplete. Molecular profiling was performed across several different next-generation sequencing platforms with differing gene coverage and analytical sensitivity, which may introduce ascertainment bias in the detection and reporting of genomic alterations. The literature review was limited by publication bias, inconsistent reporting, and variable availability of patient-level OS data. The pooled Cox analysis was exploratory, reconstructed in part from published timelines, and stratified by source to mitigate but not eliminate between-study heterogeneity. The adjusted sensitivity analysis included fewer patients and events, resulting in wide confidence intervals. Within-cohort comparisons between treatment groups were based on very small subgroups (for example, targeted-exposed versus non-exposed patients) with wide and largely overlapping confidence intervals; accordingly, the response, progression-free survival, and overall survival differences across treatment classes are descriptive only and were not formally tested for statistical significance. Therefore, the survival estimates should be interpreted as hypothesis-generating rather than definitive evidence of causality. Additional limitations include the absence of standardized criteria for treatment selection, variable timing of molecular testing, and potential misclassification inherent to retrospective pathology review across institutions and publications. The cohort also included multiple genomic subgroups that are biologically and therapeutically distinct, making pooled estimates useful for hypothesis generation but insufficient for driver-specific conclusions. Finally, because rare and favorable outcomes are more likely to be reported, the literature-derived component may overestimate benefit compared with an unselected population.

## 5. Conclusions

In conclusion, de novo AGA-positive HGNEC-L represents a rare but clinically important molecular subgroup. Collectively, the observed response patterns in the institutional cohort and published literature, directional findings of the exploratory time-dependent Cox analysis, and biological rationale support routine molecular profiling and early consideration of genotype-matched targeted therapy for patients with advanced HGNEC-L harboring de novo AGA. Prospective registry-based studies are needed to define optimal sequencing and clarify whether genotype-matched targeted therapy improves survival. Such efforts should capture baseline genomic status, histologic composition, treatment timing, radiologic response, duration of targeted exposure, mechanisms of resistance, and survival outcomes using standardized definitions. Until such evidence is available, treatment should be individualized, but the presence of a de novo AGA should be considered a clinically relevant therapeutic consideration rather than an incidental molecular finding.

## Figures and Tables

**Table 1:** (A) Baseline patient characteristics (*n* = 10). (B) Baseline tumor characteristics (*n* = 10). (C) Baseline treatment characteristics (*n* = 10).

**A. Baseline Patient Characteristic**		**Patients (*n* = 10)**	
Age, median (range), years		64.6 (47.1–77.0)	
**Gender, *n* (%)**			
Female		9 (90.0%)	
Male		1 (10.0%)	
**Smoking status, *n* (%)**			
Current/past smoker		3 (30.0%)	
Never smoker		7 (70.0%)	
Smoking exposure, median (range), pack-years		0 (0–20)	
Family history of LC, *n* (%)		0 (0.0%)	
**ECOG PS, *n* (%)**			
0		4 (40.0%)	
1		5 (50.0%)	
3		1 (10.0%)	
**B. Tumor Characteristics**		**Patients (*n* = 10)**	
**Histology, *n* (%)**			
LCNEC		6 (60.0%)	
SCLC/mixed SCLC		4 (40.0%)	
**NGS method, *n* (%)**			
Oncomine Dx		6 (60.0%)	
Tempus xT		2 (20.0%)	
FoundationOne CDx		1 (10.0%)	
Guardant360		1 (10.0%)	
**De novo AGA, *n* (%)**			
*EGFR* ex19del mut		5 (50.0%)	
*EGFR* ex20ins mut		1 (10.0%)	
*EML4-ALK* fusion		2 (20.0%)	
*KIF5B-RET* fusion		1 (10.0%)	
*KRAS* p.G12C mut		1 (10.0%)	
**Reported co-mutations, *n* (%)**			
*TP53* co-mut		6 (60.0%)	
*RB1* co-mut		3 (30.0%)	
**Biomarker data**			
PD-L1 available, *n* (%)		3 (30.0%)	
PD-L1 TPS, median (range), %		5.0 (0.0–40.0)	
TMB available, *n* (%)		4 (40.0%)	
TMB, median (range), mut/Mb		6.2 (5.6–17.1)	
MSI available, *n* (%)		4 (40.0%)	
MSI-stable, *n* (%)		4 (40.0%)	
Brain metastases at advanced disease diagnosis, *n* (%)		3 (30.0%)	
**C. Tx Characteristics**	**All Tx Lines, *n* (%)**	**Patients Ever Exposed, *n* (%)**	**1st-Line Tx, *n* (%)**
**Tx classes**			
CMT	5 (26.3%)	5 (50.0%)	2 (20.0%)
Chemo-ICI	3 (15.8%)	3 (30.0%)	3 (30.0%)
Targeted therapy	6 (31.6%)	5 (50.0%)	3 (30.0%)
Chemo-targeted therapy	3 (15.8%)	2 (20.0%)	2 (20.0%)
ICI	1 (5.3%)	1 (10.0%)	0 (0.0%)
ADC	1 (5.3%)	1 (10.0%)	0 (0.0%)
**Grouped Tx classes**			
Targeted-containing Tx	9 (47.4%)	7 (70.0%)	5 (50.0%)
ICI-containing Tx	4 (21.1%)	4 (40.0%)	3 (30.0%)
CMT only	5 (26.3%)	5 (50.0%)	2 (20.0%)
ADC	1 (5.3%)	1 (10.0%)	0 (0.0%)

**Table 2:** Real-world objective response and disease control rates by treatment class (A), grouped treatment class (B), and first-line treatment (C).

**A. Tx Classes**	**rwORR**	**rwDCR**
CMT	1/4 (25.0%)	1/4 (25.0%)
Chemo-ICI	2/2 (100.0%)	2/2 (100.0%)
Targeted therapy	4/6 (66.7%)	5/6 (83.3%)
Chemo-targeted therapy	3/3 (100.0%)	3/3 (100.0%)
ICI	0/1 (0.0%)	0/1 (0.0%)
ADC	0/1 (0.0%)	0/1 (0.0%)
**B. Grouped Tx Classes**		
Targeted-containing Tx	7/9 (77.8%)	8/9 (88.9%)
ICI-containing Tx	2/3 (66.7%)	2/3 (66.7%)
CMT only	1/4 (25.0%)	1/4 (25.0%)
ADC	0/1 (0.0%)	0/1 (0.0%)
**C. 1st-Line Tx**		
Targeted-containing Tx	3/5 (60.0%)	4/5 (80.0%)
ICI-containing Tx	2/2 (100.0%)	2/2 (100.0%)
CMT only	1/1 (100.0%)	1/1 (100.0%)

**Table 3:** Systemic treatment efficacy in HGNEC-L harboring de novo AGA.

Publication	Histology	AGA	*n*	Tx Type	Reported Outcomes	OS, mo
Heijboer et al., 2025 [12]	LCNEC	Mixed AGA (mostly *EGFR*/*ALK*)	28	Different TKIs	PR 19/38 TKIs lines	mOS—14.6 (95% CI, 11–32)
Doubre et al., 2021 [13]	LCNEC	*ALK* fusion	3	*ALK* TKIs	All 3 reportedly benefited; one prolonged SD for 56+ mo	14+, 56+, 7
Wiedemann et al., 2022 [14]	LCNEC	*EML4-ALK*	1	Crizotinib -> alectinib -> ceritinib -> brigatinib -> CMT -> lorlatinib -> chemo-ICI	PD on crizotinib, prolonged 10 mo response with alectinib, prolonged 8 mo SD with lorlatinib after ceritinib and brigatinib failure, PD on chemo-ICI	37
Kobayashi et al., 2023 [15]	LCNEC	*ALK* fusion	1	*ALK* TKIs	OS 24+ mo	24+
Tashiro et al., 2020 [16]	LCNEC	*ALK* fusion	1	Alectinib	Prolonged response for 11 mo	11+
Masuda et al., 2021 [17]	LCNEC	*ALK* fusion	1	Alectinib	Short-lived dramatic PR	4+
Leblanc et al., 2021 [18]	LCNEC	*EML4-ALK*	1	Alectinib -> brigatinib -> lorlatinib	Short-lived PR	8
Jiang et al., 2025 [19]	LCNEC	*EGFR* p.L858R	1	Osimertinib -> EP -> aumolertinib + anlotinib	PFS with targeted Tx—20 mo; 21 mo	48
Akhoundova et al., 2022 [20]	LCNEC	*EML4-ALK*	3	Alectinib, lorlatinib	2/3 patients—rapid response to alectinib; 1 progressed after 10 mo then responded to lorlatinib	22.0+, 6.0+, 10.1
Zheng et al., 2018 [21]	LCNEC	*ALK* fusion	2	Crizotinib, CMT	Cisplatin/pemetrexed with intracranial PD, crizotinib with prolonged 10+ mo response	3+, 10+
De Pas et al., 2011 [22]	LCNEC	*EGFR* ex19del	1	Gefitinib	Long-lasting response	6+
Sakai et al., 2013 [23]	LCNEC	*EGFR* p.L858R	1	CMT	CMT failure	6+
Aroldi et al., 2014 [24]	LCNEC	*EGFR* ex19del	1	CMT -> gefitinib	Clinical benefit	5.25+
Wang et al., 2015 [25]	LCNEC	*EGFR* ex19del	1	Icotinib	Radiographic response	8+
Ito et al., 2017 [26]	LCNEC + adenoca	*EGFR* p.L858R	1	*EGFR* TKIs	Radiographic response	NR
Hayashi et al., 2018 [27]	LCNEC	*ALK* fusion	1	Alectinib, CMT	Prolonged response with alectinib following progression on CMT	13+
Ricciuti et al., 2018 [28]	LCNEC	*EML4-ALK*, *MET* ex14skip	1	Crizotinib	“Lazarus response”	2+
Wang et al., 2019 [29]	LCNEC	*ALK* fusion	1	EP -> crizotinib	EP—SD for 1.5 mo, crizotinib—PR for 10 mo	11.5+
Muto et al., 2020 [30]	LCNEC + adenoca	*EGFR* ex19del	1	Afatinib -> erlotinib + bevacizumab	SD 10 mo with afatinib; SD 10 mo with erlotinib + bevacizumab	18+
Arora et al., 2023 [31]	LCNEC	*KIF5B-RET*	1	EP -> selpercatinib	Extra- and intracranial PR, ongoing benefit at 12 mo	12.7+
Chen et al., 2023 [32]	LCNEC	*EML4-ALK*	2	Alectinib	PR in both cases; PFS 21+ mo; 32+ mo	21+, 32+
Zullo et al., 2024 [33]	SCLC	*EGFR* ex20ins	2	CMT, chemo-ICI, amivantamab, *EGFR* TKIs	Prolonged PR with carboplatin/paclitaxel, failure of chemo-ICI, late-line amivantamab failure, late-line osimertinib failure; PR with chemo-ICI, late-line mobocertinib failure, late-line amivantamab failure	36, 12
Eihuku et al., 2024 [34]	LCNEC	*KIF5B-RET*	1	Selpercatinib	Durable disease control	19+
Kulkarni et al., 2025 [35]	SCLC	*BRAF* p.V600E	1	Dabrafenib + trametinib	Durable complete remission	47+
Shimizu et al., 2019 [36]	LCNEC	*KIF5B-ALK*	1	Crizotinib, alectinib	Crizotinib—SD for 10 mo, alectinib—intracranial response	24.9+
Omachi et al., 2014 [37]	LCNEC	*EML4-ALK*	1	Crizotinib	Crizotinib-resistant case	1.5+
Okamoto et al., 2006 [38]	SCLC	*EGFR* ex19del	1	Gefitinib	Gefitinib-responsive case	0.75+
Shiao et al., 2011 [39]	SCLC	*EGFR* ex19del	2	EP, topotecan, doxorubicin, gefitinib, oral etoposide	Short-lived responses to CMT; no response to gefitinib	10, 10
Batra et al., 2021 [40]	SCLC + adenoca	*EGFR* ex19del	1	EP -> osimertinib	PD after 3 cycles of EP; PR to osimertinib	9+

**Table 4:** Exploratory pooled reconstructed-IPD, source-stratified, time-dependent Cox models of OS.

Model	Pts, *n*	Deaths, *n*	Covariate	HR	95% CI	*p* Value
Primary pooled reconstructed-IPD, source-stratified, time-dependent Cox model	72	39	Targeted-Tx exposure	0.89	0.31–2.56	0.831
Exact-age + sex sensitivity subset, source-stratified, time-dependent Cox model	46	18	Targeted-Tx exposure, age, sex	0.54	0.16–1.77	0.306
	46	18	Age, per 10-year increase	0.90	0.58–1.40	0.633
	46	18	Male sex	1.17	0.28–4.96	0.828

## Data Availability

The original contributions presented in this study are included in the article/Supplementary Materials. Further inquiries can be directed to the corresponding author.

## Supplementary Materials

The following supporting information can be downloaded at: https://www.mdpi.com/article/10.3390/medsci14040491/s1, Supplementary Table S1. Individual-patient records entered into the Cox rerun.

## Abbreviations

The following abbreviations are used in this manuscript:

ADC	Antibody-Drug Conjugate
Adenoca	Adenocarcinoma
AGA	Actionable Genomic Alterations
*ALK*	Anaplastic Lymphoma Kinase
*BRAF*	B-Raf Proto-Oncogene Serine/Threonine-Protein Kinase
CI	Confidence Interval
CMT	Chemotherapy
ECOG PS	Eastern Cooperative Oncology Group Performance Status
*EGFR*	Epidermal Growth Factor Receptor
*EML4*	Echinoderm Microtubule-Associated Protein-Like 4
EP	Etoposide + Platinum-based chemotherapy
Ex19del	Exon 19 deletion
Ex20ins	Exon 20 Insertion
HGNEC-L	High-Grade Pulmonary Neuroendocrine Carcinoma
HR	Hazard Ratio
ICI	Immune Checkpoint Inhibitors
IPD	Individual Patient Data
*KIF5B*	Kinesin Family Member 5B
*KRAS*	Kirsten Rat Sarcoma viral oncogene homolog
LC	Lung Cancer
LCNEC	Large-Cell Neuroendocrine Carcinoma
*MET* ex14skip	Mesenchymal–Epithelial Transition factor gene exon 14 skipping
Mo	Months
MSI	Microsatellite Instability
(co)mut	(co)-mutation
Mut/Mb	Mutations Per Megabase
NGS	Next-Generation Sequencing
NR	Not Reported/Not Reached
(m)OS	(median) Overall Survival
PD	Progressive Disease
PD-L1	Programmed Death-Ligand 1
PFS	Progression-Free Survival
PR	Partial Response
Pts	Patients
*RB1*	Retinoblastoma 1
*RET*	Rearranged During Transfection
rwDCR	real-world Disease Control Rate
rwORR	Real-World Objective Response Rate
(m)rwPFS	(median) Real-World Progression-Free Survival
SCLC	Small-Cell Lung Cancer
SD	Stable Disease
TKIs	Tyrosine Kinase Inhibitors
TMB	Tumor Mutational Burden
*TP53*	Tumor Protein 53
TPS	Tumor Proportion Score
Tx	Treatment
