# Supplementary material for: De Novo Actionable Genomic Alterations in High-Grade Pulmonary Neuroendocrine Carcinomas: Therapeutic Implications of Targeted Treatment
Source: Med Sci (Basel). 2026 Aug 18;14(4):491. doi: 10.3390/medsci14040491 (PMC13515143; doi:10.3390/medsci14040491)
Supplement: Supplementary file 1 [file medsci-14-00491-s001.zip › medsci-4445636-supplementary.pdf]

Supplementary Materials

Supplementary Table S1. Individual-patient records entered into the pooled reconstructed-IPD, source-stratified, time-dependent Cox analysis (n=72).

Publication	Histology	Driver	Age, y	Sex	Time to targeted, mo	Time on targeted, mo	OS since 1L, mo
Akhoundova et al., 2022	LCNEC	*EML4-ALK*	37	M	0	22.0+	22.0+
Akhoundova et al., 2022	LCNEC	*EML4-ALK*	32	F	1	5.0+	6.0+
Akhoundova et al., 2022	LCNEC	*EML4-ALK*	68	F	10	0.1	10.1
Araki/Okamoto, 2005/2006	SCLC	*EGFR* exon 19 deletion	72	F	0	0.75+	0.75+
Aroldi et al., 2014	LCNEC	*EGFR* exon 19 deletion	47	F	0.25	5.0+	5.25+
Arora et al., 2023	LCNEC	*KIF5B-RET* fusion	52	F	0.7	12.0+	12.7+
Batra et al., 2021	Synchronous SCLC + adenocarcinoma	*EGFR* exon 19 deletion	39	M	3	9.0+	9.0+
Chen et al., 2023	LCNEC	*EML4-ALK*	53	F	0	21.0+	21.0+
Chen et al., 2023	LCNEC	*EML4-ALK*	62	F	0	32.0+	32.0+
De Pas et al., 2011	LCNEC	*EGFR* exon 19 p.L747_A755delinsAT	66	F	0	6.0+	6.0+
Doubre et al., 2021	LCNEC with carcinoid morphology	*ALK* rearrangement	58	F	6	8+	14.0+
Doubre et al., 2021	LCNEC with carcinoid morphology	*ALK* rearrangement	74	F	4.5	51.5+	56.0+
Doubre et al., 2021	LCNEC	*ALK* rearrangement	34	F	0	7	7
Eihuku et al., 2024	LCNEC	*KIF5B-RET* fusion	70	F	0	19.0+	19.0+
Hayashi et al., 2018	LCNEC	*ALK* rearrangement	75	F	7	6.0+	13.0+
Heijboer et al., 2025	*ALK*1	LCNEC	NR	NR	0	33	33
Heijboer et al., 2025	*ALK*2	LCNEC	NR	NR	0	7.3	7.3
Heijboer et al., 2025	*ALK*3	LCNEC	NR	NR	0	14.5+	14.5+
Heijboer et al., 2025	*ALK*4	LCNEC	NR	NR	7	8.1	15.1
Heijboer et al., 2025	*ALK*5	LCNEC	NR	NR	4.6	21.8	26.3
Heijboer et al., 2025	*ALK*6	LCNEC	NR	NR	0	9	9
Heijboer et al., 2025	*ALK*7	LCNEC	NR	NR	0	18.8	18.8
Heijboer et al., 2025	*ALK*8	LCNEC	NR	NR	0	37.1	37.1
Heijboer et al., 2025	*BRAF*1	LCNEC	NR	NR	0	40.3	40.3
Heijboer et al., 2025	*BRAF*2	LCNEC	NR	NR	0	7.3	7.3
Heijboer et al., 2025	*EGFR*1	LCNEC	NR	NR	0	78.3+	78.3+
Heijboer et al., 2025	*EGFR*2	LCNEC	NR	NR	0	12	12
Heijboer et al., 2025	*EGFR*3	LCNEC	NR	NR	3.3	12.1+	15.4+
Heijboer et al., 2025	*EGFR*4	LCNEC	NR	NR	0	15	15
Heijboer et al., 2025	*EGFR*5	LCNEC	NR	NR	0	2.2	2.2
Heijboer et al., 2025	*EGFR*6	LCNEC	NR	NR	0	33	33
Heijboer et al., 2025	*EGFR*7	LCNEC	NR	NR	10	3.4	13.4
Heijboer et al., 2025	*EGFR*8	LCNEC	NR	NR	0	11.7	11.7

Publication	Histology	Driver	Age, y	Sex	Time to targeted, mo	Time on targeted, mo	OS since 1L, mo
Heijboer et al., 2025	*EGFR*9	LCNEC	NR	NR	0	36+	36+
Heijboer et al., 2025	FGFR3	LCNEC	NR	NR	0	7.1	7.1
Heijboer et al., 2025	*KRAS*1	LCNEC	NR	NR	4.2	1.9	6.1
Heijboer et al., 2025	*KRAS*2	LCNEC	NR	NR	5.8	5.7	11.5
Heijboer et al., 2025	*KRAS*3	LCNEC	NR	NR	2.9	1.7	4.6
Heijboer et al., 2025	*RET*1	LCNEC	NR	NR	0	1.4	1.4
Heijboer et al., 2025	*RET*2	LCNEC	NR	NR	0	44.2+	44.2+
Heijboer et al., 2025	*ROS1*	LCNEC	NR	NR	0	31.2	31.2
Ito et al., 2017	LCNEC+ adenoca	*EGFR* L858R	68	F	NR	NR	NR
Jiang et al., 2025	LCNEC	*EGFR* L858R	47	F	0	48	48
Kobayashi et al., 2023	Combined LCNEC + adenocarcinoma	*ALK* rearrangement	57	F	0	24.0+	24.0+
Kulkarni et al., 2025	SCLC	*BRAF* V600E	54	M	2	45.0+	47.0+
Leblanc et al., 2021	LCNEC	*EML4-ALK*	37	M	0.5	7.5	8
Masuda et al., 2021	LCNEC	*ALK* rearrangement	72	M	0	4.0+	4.0+
Muto et al., 2020	LCNEC + adenocarcinoma	*EGFR* exon 19 deletion	70	F	0	18+	18+
Omachi et al., 2014	LCNEC	*EML4-ALK* variant 2	43	F	0	1.5+	1.5+
Our cohort	LCNEC	*EGFR* ex19del	47.1	F	5.7	7.7	13.4
Our cohort	mixed SCLC	*EGFR* ex20	74.4	F			4.8
Our cohort	LCNEC	*RET* rearrangement - *KIF5B-RET* fusion	69.6	M	0	4.4+	4.4+
Our cohort	LCNEC	*EGFR* ex19del	61.9	F			17.2
Our cohort	LCNEC	*KRAS* G12C	60.7	F			19.5
Our cohort	SCLC	*EGFR* ex19del	77	F	0	11	11
Our cohort	LCNEC	*EML4-ALK*	67.4	F	0.7	35.6	36.3
Our cohort	LCNEC	*EML4-ALK*	51.6	F	0	39.6+	39.6+
Our cohort	SCLC	EX-19 del	59	F	0	19.0	19.0
Our cohort	SCLC	EX-19 del	68.6	F	0	8.0	13.0
Ricciuti et al., 2018	LCNEC	*EML4-ALK* + *MET* exon 14 R988C	58	F	NR	2.0+	2.0+
Sakai et al., 2013	LCNEC	*EGFR* L858R	78	F	6.0+	0	6.0+
Shiao et al., 2011	SCLC	*EGFR* ex19del	63	M	10	0	10
Shiao et al., 2011	SCLC	*EGFR* ex19del	54	F	5	5	10
Shimizu et al., 2019	LCNEC	*KIF5B-ALK* fusion	73	M	0	24.9+	24.9+
Tashiro et al., 2020	LCNEC	*ALK* rearrangement	32	F	0	11	11.0+
Wang et al., 2015	LCNEC	*EGFR* exon 19 deletion	84	M	0	8	8.0+
Wang et al., 2019	LCNEC	*ALK* rearrangement	51	M	1.5	10	11.5+
Wiedemann et al., 2022	LCNEC	*EML4-ALK*	47	F	0	37	37
Zheng et al., 2018	LCNEC	*ALK* rearrangement	44	M	3	0	3+
Zheng et al., 2018	LCNEC	*EML4-ALK*	47	F	0	10.0+	10.0+

Publication	Histology	Driver	Age, y	Sex	Time to targeted, mo	Time on targeted, mo	OS since 1L, mo
Zullo et al., 2024	SCLC	*EGFR* exon 20 insertion V769_D770insASV	61	F	11	25	36
Zullo et al., 2024	SCLC	*EGFR* exon 20 insertion V769_D770insASV	30	M	8	4	12

Rows highlighted in yellow denote the present institutional cohort (n=10). "+" indicates a censored observation (patient alive at last follow-up). Overall survival (OS) is measured from initiation of first-line systemic therapy.
